# Supplementary material for: The rate of epigenetic drift scales with maximum lifespan across mammals
Source: Nat Commun. 2023 Nov 25;14:7731. doi: 10.1038/s41467-023-43417-6 (PMC10676422; doi:10.1038/s41467-023-43417-6)
Supplement: Supplementary file 1 — Supplementary Information [file 41467_2023_43417_MOESM1_ESM.pdf]

The rate of epigenetic drift scales with maximum lifespan across mammals

Supplemental Tables

Table S1: Gene ontology results from regions with age-associated disorder in rats. Top 20 hits from molecular function (MF) and biological process (BP) are shown. Enrichment tests were done using Fisher's one-tailed test in g:Profiler using a custom background of all the genes which were represented in the rat dataset. P-values were adjusted for multiple testing using the default g:SCS.

Source	GO Term Name	GO ID	Adjusted p-value
GO:MF	DNA-binding transcription factor activity	GO:0003700	2.98E-13
GO:MF	DNA-binding transcription factor activity, RNA polymerase II-specific	GO:0000981	1.41E-12
GO:MF	sequence-specific double-stranded DNA binding	GO:1990837	1.48E-11
GO:MF	sequence-specific DNA binding	GO:0043565	2.08E-11
GO:MF	transcription regulator activity	GO:0140110	4.62E-11
GO:MF	double-stranded DNA binding	GO:0003690	5.34E-11
GO:MF	transcription cis-regulatory region binding	GO:0000976	1.47E-10
GO:MF	transcription regulatory region nucleic acid binding	GO:0001067	1.95E-10
GO:MF	RNA polymerase II transcription regulatory region sequence-specific DNA binding	GO:0000977	5.09E-10
GO:MF	cis-regulatory region sequence-specific DNA binding	GO:0000987	5.36E-10
GO:MF	RNA polymerase II cis-regulatory region sequence-specific DNA binding	GO:0000978	6.68E-10

GO:MF	protein binding	GO:0005515	2.92E-09
GO:MF	binding	GO:0005488	6.48E-08
GO:MF	DNA-binding transcription activator activity	GO:0001216	3.5552E-06
GO:MF	DNA-binding transcription activator activity, RNA polymerase II-specific	GO:0001228	7.6306E-06
GO:MF	molecular function	GO:0003674	1.8436E-05
GO:MF	DNA binding	GO:0003677	1.8534E-05
GO:MF	nucleoside-triphosphatase regulator activity	GO:0060589	0.00011267
GO:MF	GTPase regulator activity	GO:0030695	0.00011267
GO:MF	guanyl-nucleotide exchange factor activity	GO:0005085	0.00026922
GO:BP	nervous system development	GO:0007399	2.06E-35
GO:BP	anatomical structure morphogenesis	GO:0009653	1.06E-34
GO:BP	neurogenesis	GO:0022008	4.38E-33
GO:BP	multicellular organism development	GO:0007275	4.67E-32
GO:BP	system development	GO:0048731	2.26E-31
GO:BP	cell differentiation	GO:0030154	4.87E-31
GO:BP	cellular developmental process	GO:0048869	1.09E-30
GO:BP	generation of neurons	GO:0048699	1.17E-30
GO:BP	anatomical structure development	GO:0048856	2.79E-30
GO:BP	neuron differentiation	GO:0030182	1.95E-29
GO:BP	multicellular organismal process	GO:0032501	2.19E-29
GO:BP	developmental process	GO:0032502	2.27E-28
GO:BP	cell development	GO:0048468	1.07E-26

GO:BP	neuron development	GO:0048666	2.01E-21
GO:BP	cell morphogenesis	GO:0000902	1.02E-20
GO:BP	cell morphogenesis involved in differentiation	GO:0000904	5.40E-19
GO:BP	neuron projection development	GO:0031175	3.36E-18
GO:BP	central nervous system development	GO:0007417	6.49E-18
GO:BP	regulation of developmental process	GO:0050793	2.49E-17
GO:BP	animal organ development	GO:0048513	3.61E-17

Table S2: Gene ontology results from regions with age-associated disorder in mice. Top 20 hits from molecular function (MF) and biological process (BP) are shown. Enrichment tests were done using Fisher's one-tailed test in g:Profiler using a custom background of all the genes which were represented in the mouse dataset. P-values were adjusted for multiple testing using the default g:SCS.

Source	GO Term Name	GO ID	Adjusted p-value
GO:MF	DNA-binding transcription factor activity	GO:0003700	5.78E-32
GO:MF	DNA-binding transcription factor activity, RNA polymerase II-specific	GO:0000981	8.18E-31
GO:MF	protein binding	GO:0005515	2.73E-29
GO:MF	transcription regulator activity	GO:0140110	4.14E-25
GO:MF	binding	GO:0005488	5.12E-25
GO:MF	RNA polymerase II transcription regulatory region sequence-specific DNA binding	GO:0000977	1.56E-24
GO:MF	sequence-specific double-stranded DNA binding	GO:1990837	1.57E-24
GO:MF	RNA polymerase II cis-regulatory region sequence-specific DNA binding	GO:0000978	3.13E-23

GO:MF	cis-regulatory region sequence-specific DNA binding	GO:0000987	3.38E-23
GO:MF	transcription cis-regulatory region binding	GO:0000976	7.03E-23
GO:MF	transcription regulatory region nucleic acid binding	GO:0001067	1.40E-22
GO:MF	sequence-specific DNA binding	GO:0043565	1.95E-22
GO:MF	double-stranded DNA binding	GO:0003690	2.18E-21
GO:MF	DNA-binding transcription activator activity, RNA polymerase II-specific	GO:0001228	9.26E-19
GO:MF	DNA-binding transcription activator activity	GO:0001216	1.71E-18
GO:MF	gated channel activity	GO:0022836	3.01E-16
GO:MF	voltage-gated channel activity	GO:0022832	5.32E-14
GO:MF	voltage-gated ion channel activity	GO:0005244	1.40E-13
GO:MF	voltage-gated cation channel activity	GO:0022843	5.54E-12
GO:MF	ion channel activity	GO:0005216	2.49E-11
GO:BP	anatomical structure morphogenesis	GO:0009653	3.58E-99
GO:BP	anatomical structure development	GO:0048856	2.30E-94
GO:BP	nervous system development	GO:0007399	2.71E-94
GO:BP	multicellular organism development	GO:0007275	9.33E-93
GO:BP	system development	GO:0048731	9.58E-93
GO:BP	developmental process	GO:0032502	8.87E-90
GO:BP	multicellular organismal process	GO:0032501	1.44E-83
GO:BP	neurogenesis	GO:0022008	1.36E-74

GO:BP	cellular developmental process	GO:0048869	4.89E-71
GO:BP	cell differentiation	GO:0030154	4.98E-71
GO:BP	animal organ development	GO:0048513	1.10E-67
GO:BP	generation of neurons	GO:0048699	3.00E-67
GO:BP	neuron differentiation	GO:0030182	4.57E-65
GO:BP	animal organ morphogenesis	GO:0009887	1.65E-64
GO:BP	cell development	GO:0048468	5.62E-61
GO:BP	signaling	GO:0023052	9.01E-59
GO:BP	cell communication	GO:0007154	3.56E-58
GO:BP	tissue development	GO:0009888	9.68E-56
GO:BP	cell-cell signaling	GO:0007267	6.04E-52
GO:BP	regulation of cellular process	GO:0050794	2.49E-51

Table S3: Gene ontology results from regions with age-associated disorder in dogs. Top 20 hits from molecular function (MF) and biological process (BP) are shown. Enrichment tests were done using Fisher's one-tailed test in g:Profiler using a custom background of all the genes which were represented in the dog dataset. P-values were adjusted for multiple testing using the default g:SCS.

Source	GO Term Name	GO ID	Adjusted p-value
GO:MF	DNA-binding transcription factor activity	GO:0003700	4.28E-47
GO:MF	sequence-specific DNA binding	GO:0043565	1.01E-39
GO:MF	DNA-binding transcription factor activity, RNA polymerase II-specific	GO:0000981	3.08E-39
GO:MF	transcription regulator activity	GO:0140110	1.69E-37
GO:MF	sequence-specific double-stranded DNA binding	GO:1990837	1.08E-36

GO:MF	double-stranded DNA binding	GO:0003690	6.22E-34
GO:MF	DNA binding	GO:0003677	3.01E-30
GO:MF	transcription cis-regulatory region binding	GO:0000976	3.33E-17
GO:MF	transcription regulatory region nucleic acid binding	GO:0001067	3.86E-17
GO:MF	RNA polymerase II transcription regulatory region sequence-specific DNA binding	GO:0000977	7.91E-16
GO:MF	cis-regulatory region sequence-specific DNA binding	GO:0000987	1.55E-15
GO:MF	nucleic acid binding	GO:0003676	5.18E-15
GO:MF	RNA polymerase II cis-regulatory region sequence-specific DNA binding	GO:0000978	5.59E-13
GO:MF	DNA-binding transcription activator activity, RNA polymerase II-specific	GO:0001228	1.15E-09
GO:MF	DNA-binding transcription activator activity	GO:0001216	2.19E-09
GO:MF	organic cyclic compound binding	GO:0097159	1.42E-07
GO:MF	heterocyclic compound binding	GO:1901363	2.05E-07
GO:MF	binding	GO:0005488	6.83E-07
GO:MF	DNA-binding transcription repressor activity	GO:0001217	1.7135E-06
GO:MF	DNA-binding transcription repressor activity, RNA polymerase II-specific	GO:0001227	4.5071E-06
GO:BP	multicellular organism development	GO:0007275	3.81E-33
GO:BP	animal organ morphogenesis	GO:0009887	5.08E-30

GO:BP	system development	GO:0048731	2.13E-29
GO:BP	anatomical structure development	GO:0048856	7.69E-28
GO:BP	anatomical structure morphogenesis	GO:0009653	5.25E-27
GO:BP	animal organ development	GO:0048513	1.05E-26
GO:BP	embryo development	GO:0009790	1.43E-26
GO:BP	regulation of transcription, DNA-templated	GO:0006355	2.06E-26
GO:BP	regulation of nucleic acid-templated transcription	GO:1903506	2.46E-26
GO:BP	regulation of RNA biosynthetic process	GO:2001141	3.85E-26
GO:BP	multicellular organismal process	GO:0032501	6.84E-26
GO:BP	regulation of gene expression	GO:0010468	2.64E-25
GO:BP	regulation of RNA metabolic process	GO:0051252	1.29E-24
GO:BP	embryonic morphogenesis	GO:0048598	1.80E-24
GO:BP	transcription, DNA-templated	GO:0006351	2.11E-24
GO:BP	nucleic acid-templated transcription	GO:0097659	2.50E-24
GO:BP	RNA biosynthetic process	GO:0032774	2.70E-24
GO:BP	developmental process	GO:0032502	5.93E-24
GO:BP	embryonic organ morphogenesis	GO:0048562	3.61E-22
GO:BP	regulation of nucleobase-containing compound metabolic process	GO:0019219	7.22E-22

Table S4: Gene ontology results from regions with age-associated disorder in baboons. Hits from human phenotypes (HP) are shown. Enrichment tests were done using Fisher's one-tailed test in g:Profiler using a custom background of all the genes which were represented in the baboon dataset. P-values were adjusted for multiple testing using the default g:SCS.

Source	GO Term Name	GO ID	Adjusted p-value
HP	Tortuous cerebral arteries	HP:0004938	0.0012038
HP	Arterial tortuosity	HP:0005116	0.02200196
HP	Vascular tortuosity	HP:0004948	0.03636136
