# Supplementary material for: The rate of epigenetic drift scales with maximum lifespan across mammals
Source: Nat Commun. 2023 Nov 25;14:7731. doi: 10.1038/s41467-023-43417-6 (PMC10676422; doi:10.1038/s41467-023-43417-6)
Supplement: Supplementary file 3 — Reporting Summary [file 41467_2023_43417_MOESM3_ESM.pdf]

nature portfolio

Corresponding author(s): Benjamin B. Parrott, Emily M. Bertucci-Richter

Last updated by author(s): 10-26-2023

Reporting Summary

Nature Portfolio wishes to improve the reproducibility of the work that we publish. This form provides structure for consistency and transparency in reporting. For further information on Nature Portfolio policies, see our Editorial Policies and the Editorial Policy Checklist.

Statistics

For all statistical analyses, confirm that the following items are present in the figure legend, table legend, main text, or Methods section.

n/a	Confirmed	
☐	☒	The exact sample size (n) for each experimental group/condition, given as a discrete number and unit of measurement
☐	☒	A statement on whether measurements were taken from distinct samples or whether the same sample was measured repeatedly
☐	☒	The statistical test(s) used AND whether they are one- or two-sided<br>*Only common tests should be described solely by name; describe more complex techniques in the Methods section.*
☐	☒	A description of all covariates tested
☐	☒	A description of any assumptions or corrections, such as tests of normality and adjustment for multiple comparisons
☐	☒	A full description of the statistical parameters including central tendency (e.g. means) or other basic estimates (e.g. regression coefficient) AND variation (e.g. standard deviation) or associated estimates of uncertainty (e.g. confidence intervals)
☐	☒	For null hypothesis testing, the test statistic (e.g. F, t, r) with confidence intervals, effect sizes, degrees of freedom and P value noted<br>*Give P values as exact values whenever suitable.*
☒	☐	For Bayesian analysis, information on the choice of priors and Markov chain Monte Carlo settings
☒	☐	For hierarchical and complex designs, identification of the appropriate level for tests and full reporting of outcomes
☐	☒	Estimates of effect sizes (e.g. Cohen's d, Pearson's r), indicating how they were calculated

Our web collection on statistics for biologists contains articles on many of the points above.

Software and code

Policy information about availability of computer code

Data collection	No software or code was used for data collection.
Data analysis	Trim Galore!_v0.6.5 : Bioinformatics tool used for trimming of RRBS reads<br>Bismark_v0.22.3 : Bioinformatics tool used for alignment of RRBS reads<br>Bedtools_v2.26.0 : Bioinformatics tool used for manipulation of genomic regions<br>R_v3.6.1 : R Programing language used for statistical analyses<br>R_psych_v2.1.9 : R package used for Pearson's correlations<br>R_lme4_v1.1-27.1 : R package used to fit linear models<br>R_lmerTest_v3.1-3 : R package used to estimate p-values from linear mixed effects models<br>Galaxy_v1.3.1 : web tool used to calculate CpG density of bed file coordinates<br>gProfiler : web tool used for gene ontology enrichment analyses<br>Examples of custom R scripts used for regional disorder measurement are available at: https://github.com/embertucci/epigenetic-disorder

For manuscripts utilizing custom algorithms or software that are central to the research but not yet described in published literature, software must be made available to editors and reviewers. We strongly encourage code deposition in a community repository (e.g. GitHub). See the Nature Portfolio guidelines for submitting code & software for further information.

Data

Policy information about availability of data

All manuscripts must include a data availability statement. This statement should provide the following information, where applicable:

- Accession codes, unique identifiers, or web links for publicly available datasets
- A description of any restrictions on data availability
- For clinical datasets or third party data, please ensure that the statement adheres to our policy

The data that support the findings of this study are openly available in NCBI's Sequence Read Archive under BioProject IDs: PRJNA319643 [https://www.ncbi.nlm.nih.gov/bioproject/PRJNA319643], PRJNA675651 [https://www.ncbi.nlm.nih.gov/bioproject/PRJNA675651], PRJNA612432 [https://www.ncbi.nlm.nih.gov/bioproject/PRJNA612432] and PRJNA648767 [https://www.ncbi.nlm.nih.gov/bioproject/PRJNA648767]. Maximum lifespan estimates were collected from AnAge Database (https://genomics.senescence.info/species/index.html).

Research involving human participants, their data, or biological material

Policy information about studies with human participants or human data. See also policy information about sex, gender (identity/presentation), and sexual orientation and race, ethnicity and racism.

Reporting on sex and gender	Sex specific analyses were not performed due to unequal representation of females across species.
Reporting on race, ethnicity, or other socially relevant groupings	This information has not been collected.
Population characteristics	This information has not been collected.
Recruitment	We used previously generated data and thus there was no recruitment done for this study.
Ethics oversight	We used previously generated data and thus there was no ethics oversight.

Note that full information on the approval of the study protocol must also be provided in the manuscript.

Field-specific reporting

Please select the one below that is the best fit for your research. If you are not sure, read the appropriate sections before making your selection.

[x] Life sciences [ ] Behavioural & social sciences [ ] Ecological, evolutionary & environmental sciences

For a reference copy of the document with all sections, see nature.com/documents/nr-reporting-summary-flat.pdf

Life sciences study design

All studies must disclose on these points even when the disclosure is negative.

Sample size	We utilized reduced representation bisulfite sequencing (RRBS) data derived from the whole blood of male mice (Mus musculus; n = 153; BioProject ID: PRJNA319643), male rats (Rattus norvegicus; n = 134; BioProject ID: PRJNA675651), male and female dogs (Canis lupus familiaris; n = 107; BioProject ID: PRJNA612432), and male and female baboons (Papio cynocephalus; n = 250; BioProject ID: PRJNA648767).
Data exclusions	Longitudinal samples in the baboon dataset were removed from all analyses to prevent pseudo-replication (final n = 212).
Replication	We analyzed the relationship between epigenetic disorder and age at several scales of genomic resolution - ranging from 200 base pair regions to genome wide averages.We show robust evidence that epigenetic disorder is associated with age across all levels of genomic organization.
Randomization	This study was an observational in nature and thus randomization was not relevant.
Blinding	This study was an observational in nature and thus blinding was not relevant.

Reporting for specific materials, systems and methods

We require information from authors about some types of materials, experimental systems and methods used in many studies. Here, indicate whether each material, system or method listed is relevant to your study. If you are not sure if a list item applies to your research, read the appropriate section before selecting a response.

Materials & experimental systems

n/a	Involved in the study
☒	☐ Antibodies
☒	☐ Eukaryotic cell lines
☒	☐ Palaeontology and archaeology
☒	☐ Animals and other organisms
☒	☐ Clinical data
☒	☐ Dual use research of concern
☒	☐ Plants

Methods

n/a	Involved in the study
☒	☐ ChIP-seq
☒	☐ Flow cytometry
☒	☐ MRI-based neuroimaging
